# Innovative Strategies to Decompose Pollutants

**DOI:** 10.3390/toxics13070569

**Published:** 2025-07-05

**Authors:** Fabrizio Olivito, Pravin Jagdale

**Affiliations:** 1 Department of Environmental Engineering, University of Calabria, Via P. Bucci, 87036 Arcavacata di Rende, CS, Italy; 2 BHUMI-Bharat harit urja Managment and Innovations Pvt Ltd., Bansenstrasse, 21075 Hamburg, Germany; pravin.jagdale@circular-carbon.com

In an era marked by growing environmental awareness and a global commitment to achieving carbon neutrality, many human activities continue to contribute significantly to the pollution of water, soil, and air through the release of both organic and inorganic contaminants [1]. To address this issue, numerous strategies have been developed, many of which focus on the use of absorption and adsorption techniques employing both natural and synthetic materials [2,3]. Synthetic polymers—such as polyurethanes, microgels, and resins—are widely used across various sectors due to their advantageous physical and chemical properties, which make them effective in capturing and removing pollutants from different environments [4,5]. In addition, although they constitute materials synthesized from synthetic resources, today, several green technologies, using renewable or natural substances, are employed, with comparable or even superior effects and properties [6]. Concurrently, increasing attention is being paid to natural alternatives [7]. Lignocellulosic materials, fungal biomass, agricultural waste, and other organic substances have demonstrated remarkable potential as sustainable and cost-effective absorbents [8]. These materials not only contribute to pollutant removal but also align with circular economy principles by valorizing waste products [9]. Absorption, however, is not the only method currently being explored for pollution mitigation. Decomposition and degradation techniques—whether chemical, biological, or photolytic—also offer promising pathways for the breakdown of harmful compounds into less toxic or inert forms, further contributing to environmental remediation efforts. A wide range of harmful substances are used or released in high-impact sectors. Pesticides in agriculture, combustion gases from industrial processes, synthetic dyes from the textile industry, pharmaceutical residues, and many other chemical agents continue to pose a serious threat to ecosystems and human health [10,11]. The development and implementation of innovative, efficient, and sustainable methods for their detection, capture, and neutralization remain critical challenges in the pursuit of a cleaner and more resilient environment. Degrading substances harmful to the ecosystem into harmless or less toxic compounds represents one of the most promising frontiers in research efforts to combat environmental pollution. This approach, often referred to as advanced degradation or remediation, seeks not merely to remove pollutants but to transform them into substances that no longer pose a risk to living organisms or ecosystems. Catalysis plays a pivotal role in this process, both from a chemical and biological standpoint. Chemical catalysis, including photocatalysis and redox catalysis, facilitates the acceleration of degradation reactions under controlled conditions, often using light, heat, or specific reagents to break down persistent pollutants [12]. Materials such as titanium dioxide (TiO_2_), doped metal oxides, and engineered nanocatalysts have shown exceptional promise in this field due to their high reactivity and reusability [13]. Conversely, biocatalysis—employing enzymes or whole microorganisms—offers a more environmentally friendly alternative, often operating under milder conditions and with greater substrate specificity. Certain bacteria and fungi are capable of metabolizing complex organic pollutants, such as polycyclic aromatic hydrocarbons (PAHs), pesticides, and pharmaceutical residues, converting them into inert or biodegradable products [14]. The synergy between chemical and biological catalysis is also being explored, leading to hybrid systems wherein, for example, photocatalytic materials enhance the activity of microbial communities or enzymes are immobilized on synthetic supports to improve their stability and reusability. Moreover, these catalytic approaches are increasingly being integrated into scalable technologies, such as catalytic reactors, filtration membranes, and wastewater treatment systems, enabling their application in real-world environmental remediation scenarios [15]. As research continues to evolve, the challenge remains to develop catalytic systems that are not only efficient and selective but also economically viable and sustainable over the long term. The integration of these systems into existing industrial and municipal infrastructure is a key step toward achieving meaningful reductions in pollution and advancing the global transition toward a greener future.

As part of the following Special Issue, we have collated important scientific advancements in the field of pollutant decomposition and decontamination. Nina Petrovičová and colleagues (Contribution 1) investigated banknotes as a source of drug and pharmaceutical contamination in their study population. They developed an insightful study regarding the exposure of cashiers to micropollutants in the Slovak Republic. By handling bank notes, staff come into direct contact with traces of not only antibiotics but also common drugs. The presence of these contaminants through hand washing alone and water analysis confirms the significant presence and the concern regarding how these substances can easily enter the ecosystem through the discharge of water and contaminate the environment. Many substances are, in fact, toxic to plant and animal organisms. In another study, Mengnan Shen and colleagues (Contribution 2) reported the occurrence and health risk assessment of sulfonamide antibiotics in different freshwater fish in northeast China. Taking 12 types of antibiotics as a reference, quantitative HPLC-MS/MS analysis of the fish samples was performed. The results demonstrated that average levels of sulfonamide antibiotics in fish samples from Harbin, Changchun, and Shenyang amounted to 1.83 ng/g ww, 0.98 ng/g ww, and 1.60 ng/g ww, respectively. Sulfamethoxazole displayed the highest levels; in comparison, sulfapyridine exhibited the lowest concentrations in all of the fish samples. The concentration of antibiotics exhibited variation from city to city; however, the highest levels were found in carnivorous fish types. The results of this study demonstrate that the levels of pollutants possibly indirectly absorbed by the study population are still within the limits established by law. Fabrizio Olivito and colleagues (Contribution 3) reported an interesting synthesis of bio-based polyurethane foams produced from the renewable chemicals L-Lysine Ethyl Ester Diisocyanate (L-LDI) and Bis-hydroxymethyl Furan (BHMF). Surprisingly, the PU foams exhibited rapid biodegradation under natural conditions in soil, proving that renewable chemicals can be effective in producing biodegradable materials. The results of this study open new avenues for the synthesis of PU foams that can be easily biodegraded at the end of their life. These renewable chemicals introduced into the polymer chain have been proven to possess functional groups capable of being attacked by enzymes, organisms, and chemicals, thus producing a finished material that can be easily disposed of. Pooja Kumari et al. (Contribution 4) conducted an innovative synthesis of mixed-phase TiO_2_–ZrO_2_ nanocomposite for photocatalytic wastewater treatment. Initially, TiO_2_ nanoparticles were synthesized using a sol–gel process and ZrO_2_ was prepared using a solution combustion process. Thereafter, mixed-phase TiO_2_–ZrO_2_ nanoparticles were synthesized using the same sol–gel process to decompose Eosin Yellow (EY) from water samples. Compared to TiO_2_ and ZrO_2_, tested separately, the mixed-phase TiO_2_–ZrO_2_ nanocomposite demonstrated the best performance in terms of degradation rate and efficiency. In another paper by Shuhai Sun and colleagues (Contribution 5), the authors described a molecular design and mechanism analysis of phthalic acid ester substitutes: improved biodegradability in processes of sewage treatment and soil remediation. Phthalic acid esters (PAEs) are characterized by a low biodegradation rate and are persistent organic pollutants. In this study, the scoring function values of PAEs docking with various degradation enzymes in sewage treatment were analyzed, and a 3D quantitative structure–activity relationship (3D-QSAR) model for PAE biodegradability was developed. Following the investigation, 38 PAE substitutes were created, and the modification mechanism of PAE substitutes suitable for sewage treatment and soil environment degradation was analyzed to improve biodegradability. Lastly, in a fascinating review article by Mengnan Shen and colleagues (Contribution 6), the authors investigated the occurrence, bioaccumulation, metabolism, and ecotoxicity of fluoroquinolones in the aquatic environment. Fluoroquinolones (FQs) are among the most widespread antibiotics detected in water bodies. They are persistent organic pollutants with relevant toxicity to the ecosystem. In the review, the authors reported a comprehensive analysis of the most recent and relevant literature on FQ water pollution, reporting contamination levels of FQs in global surface water over the past three years, in addition to the bioaccumulation and metabolism patterns of FQs in aquatic organisms, their ecological toxicity, and influencing factors.
